# Nocturnal Enuresis

**Published:** 1905-10-28

**Authors:** 


### NOCTURNAL ENURESIS.

Dr. Thursfield,[1] in a lecture upon this frequent disorder, observes that in early infancy the evacuation of the bladder is a purely reflex act. A slight stimulation of the cutaneous nerves is quite sufficient to excite the bladder to contractions—a fact often emphasised in the out-patient room, where the simple exposure of an infant is promptly answered by the act of micturition. This reflex activity persists throughout the first year of life, but should be under reasonably good control by the arrival of the second birthday. Lapses occur during the third year, but anything like frequent incontinence of urine at this age must be regarded as pathological.

The bulk of cases occur between the ages of three and ten. Dr. Thursfield has found that girls preponderate in his experience of the condition, although many authorities consider its incidence evenly divided between the sexes. Control of the bladder in such children is usually efficient during the day, but occasionally cases occur in which even during waking hours the call to micturition must be answered without delay if involuntary evacuation of the bladder is to be avoided.

Of the causes, one which is likely to escape notice, unless it be suspected, is the occurrence of nocturnal epilepsy, either in its major or minor forms. This limitation of epilepsy to the sleeping hours is not common, but it may occur, and may have no other expression than incontinence of urine; it must therefore be borne in mind, for bromide therapy will sometimes succeed at once where the usual methods have resulted in prolonged failure. The author enters a warning against the frequent acceptance of enuresis as evidence of mental deficiency. He observes that in backward children the infantile condition of micturition—that is to say, the readiness of the bladder to be evacuated upon slight stimulation—may persist even in the fourth year without being an indication of cerebral defect.

Diseases of the spinal cord are in practice but seldom the cause of nocturnal enuresis; compression of the lumbar cord, due to spinal caries, is a competent source of incontinence by overflow, but is not entitled to be considered a cause of enuresis as commonly encountered. Obvious diseases of the bladder, such as stone, tuberculosis, new growths, and cystitis of whatever origin, may also be the underlying causes of incontinence, but the disease in its most frequent form has no objective pathology. It is a reflex hyperæsthesia of the centres controlling micturition, and the common stimulants at work are, in Dr. Thursfield's opinion, the following:—

First in order of importance come adenoid vegetations in the naso-pharynx. Without offering an explanation of the mechanism of this connection, the author records the fact that of twenty-five consecutive cases in his practice all were mouth-breathers. Not all of them presented this condition to a degree necessitating operation, but in those that did not, the inauguration of regular breathing exercises sufficed to remove both the mouth breathing and the enuresis in a considerable number of cases. Secondly, the presence of thread-worms in the rectum. Next vulvitis, balanitis, and phimosis. Hyperacidity of the urine also, which is a rare but sufficient cause. The author recounts the case of a boy who had suffered from lack of control over his bladder since infancy, and who differed in some respects from the type usually associated with enuresis. Thus, his incontinence was worse by day than by night; he was not excitable, but rather inert and stolid. His urine was highly acid and deposited large quantities of urates. This boy was completely cured, up to the time of writing, by the administration of potassium citrate to reduce the acidity of his urine. Bacteria present in the urine, even in the absence of symptomatic cystitis, will sometimes produce enuresis. This variety occurs after typhoid fever, but the bacteriuria may, and frequently does, depend upon other organisms than the typhoid bacillus.

But it is important to recognise that hardly any one of these causes is capable of operation unless the soil is favourable. That is to say, the basis of the trouble is some nervous instability.

As regards treatment, it is recommended that, however high the degree of immediate success, it is important to continue treatment for several months before claiming a cure, for relapse is common.

Secondly, the force of habit is so strong that the greatest effort should be made to establish a habit favourable to success. For instance, some children fall into the custom of answering the least call of the bladder for evacuation, with the result that, even when any exciting cause has been satisfactorily removed, the surviving habit leads to spontaneous micturition night after night. It is well, therefore, to enjoin the most rigid regularity of habit as regards micturition during the day. The child should be encouraged to retain his water as long as possible. He should empty his bladder before going to bed, and be waked to empty it again within an hour or so, for enuresis occurs most commonly during the first two hours of sleep. All articles of food which act as irritants or diuretics should be forbidden. Dr. Thursfield proscribes tea, coffee, sugar, and sweets generally. He does not consider it necessary to curtail the quantity of fluid swallowed, but reduces the allowance taken with the last meal of the day. All recognisable sources of peripheral irritation, such as those detailed above, require attention and removal if possible. Touching drugs, the author is of opinion that belladonna is still the stand-by for the condition, and administers it in somewhat heroic doses. He is accustomed to begin the treatment with ten minims of the tincture thrice daily, raising the quantity rapidly to thirty or forty minims. The danger of poisoning is very slight, though visual accommodation may be interfered with, and some dryness of the throat may be experienced. In the case of excitable children the addition of bromide of potassium to the draught affords a useful aid, while any indication for the administration of iron, arsenic, and tonics generally should be appropriately met. Where the urine is highly acid, citrate of potash in fifteen-grain doses will be generally successful, while in cases following an acute infectious fever, such as typhoid, urotropin in ten-grain doses well diluted has rewarded the author with considerable success.

The prognosis as illustrated by Dr. Thursfield's experience is as follows: 70 to 80 per cent. of his cases were cured in three months or less; 10 to 15 per cent. were cured in from six to eight months; and the remainder, with the exception of about ½ per cent., within the expiry of two years from the inception of the treatment.

[1] Clin. Jour., Oct. 11, 1905.

